# Influence of Cognitive Functioning on Age-Related Performance Declines in Visuospatial Sequence Learning

**DOI:** 10.3389/fpsyg.2017.00919

**Published:** 2017-06-02

**Authors:** Melanie Krüger, Mark R. Hinder, Rohan Puri, Jeffery J. Summers

**Affiliations:** ^1^Department of Sport and Health Sciences, Technical University of MunichMunich, Germany; ^2^Sensorimotor Neuroscience and Ageing Laboratory, School of Medicine, Faculty of Health, University of Tasmania, HobartTAS, Australia; ^3^Research Institute for Sport and Exercise Sciences, Liverpool John Moores UniversityLiverpool, United Kingdom

**Keywords:** factor analysis, information processing, problem solving, perceptual learning, memory, aging

## Abstract

**Objectives:** The aim of this study was to investigate how age-related performance differences in a visuospatial sequence learning task relate to age-related declines in cognitive functioning.

**Method:** Cognitive functioning of 18 younger and 18 older participants was assessed using a standardized test battery. Participants then undertook a perceptual visuospatial sequence learning task. Various relationships between sequence learning and participants’ cognitive functioning were examined through correlation and factor analysis.

**Results:** Older participants exhibited significantly lower performance than their younger counterparts in the sequence learning task as well as in multiple cognitive functions. Factor analysis revealed two independent subsets of cognitive functions associated with performance in the sequence learning task, related to either the processing and storage of sequence information (first subset) or problem solving (second subset). Age-related declines were only found for the first subset of cognitive functions, which also explained a significant degree of the performance differences in the sequence learning task between age-groups.

**Discussion:** The results suggest that age-related performance differences in perceptual visuospatial sequence learning can be explained by declines in the ability to process and store sequence information in older adults, while a set of cognitive functions related to problem solving mediates performance differences independent of age.

## Introduction

In everyday life, we often perceive and perform complex actions as a sequence of single actions. The learning of such perceptual and motor sequences is a cognitive process, in which single sequence components and their relation to each other have to be identified, processed and stored in memory (Clegg et al., 1998; Verwey et al., 2015). Since the seminal work on sequence learning using the serial reaction time-task (SRT-task) by Nissen and Bullemer (1987), sequence learning has been widely investigated. The outcome of this research has shaped our understanding of sequence learning at both the behavioral and neurophysiological level (Sakai et al., 1998; Ghilardi et al., 2003; Moisello et al., 2009; Panzer et al., 2009), influencing theory building in cognitive psychology and behavioral neuroscience (Hikosaka et al., 1999; Verwey et al., 2015).

A common finding across sequence learning studies involving cohorts of younger and older adults is the substantial difference between age groups in absolute task performance and performance improvements throughout the learning process. Older adults are consistently found to perform worse in explicit sequence learning tasks than younger adults, a finding often attributed to age-related declines in cognitive functioning (Ghilardi et al., 2003; Bo et al., 2009; Panzer et al., 2011). A number of studies have attempted to directly relate differences in sequence learning performance between younger and older adults to age-related declines in cognitive functioning. Bo, Seidler and colleagues (Bo and Seidler, 2009; Bo et al., 2009), for example, observed that age-related performance decreases in an explicit motor sequence learning task was correlated with age-related declines in visuospatial memory capacity. Similarly, Ghisletta et al. (2010) were able to show that the interaction between age and spatial working memory capacity explained around 22% of the observed inter-individual performance variability in a perceptual motor learning task. Thus, empirical evidence indicates a close relationship between age-related decrements in perceptual and motor sequence learning and age-related declines in visuospatial working memory capacity.

Other cognitive functions, such as reasoning and attention, have also been postulated to influence perceptual and motor learning. Unsworth and Engle (2005), for example, were able to relate the degree of learning of younger adults in an explicit SRT-task to the higher cognitive function of reasoning. However, while reasoning is known to be negatively impacted by aging (Horn and Cattell, 1967; Salthouse, 2005), the relationship between age-related decrements in sequence learning performance and age-related declines in reasoning ability has not been previously investigated. In contrast, age-related performance differences in explicit sequence learning tasks have already been linked to age-related declines in attentional processes, such as, e.g., the accurate allocation of attention (e.g., Clegg et al., 1998; Ghilardi et al., 2003; Mishra et al., 2015). Both, attention and spatial reasoning are also known to be related to working memory capacity (Engle et al., 1999; Glisky, 2007; Gazzaley, 2011; Ikkai and Curtis, 2011). In line with the above, age-related decrements in reasoning ability have been attributed to age-related declines in working memory capacity (Salthouse, 2005). However, the interrelation of these cognitive functions has not been accounted for in previous studies on age-related performance decrements in sequence learning.

In sum, previous studies on perceptual and motor sequence learning have revealed significant differences in learning performance between younger and older adults. A limited number of studies were able to partially explain these differences by way of age-related declines in single cognitive functions, mainly working memory capacity (Bo and Seidler, 2009; Bo et al., 2009; Ghisletta et al., 2010). The influence of other cognitive functions, such as reasoning and attention, on sequence learning performance has been previously suggested but their reciprocal interrelations and their interaction with age has not been systematically studied in the context of a learning task. Thus, the aim of this study was to investigate the influence and interaction of multiple cognitive abilities on performance differences in explicit perceptual sequence learning between younger and older adults. The level of education of participants was not considered as a variable in the current study, as previous research on implicit sequence learning reported age-related performance decrements even between younger and highly educated older adults (Howard et al., 2004). We thus assume that the educational level did not represent an explanatory factor for the expected age-group differences in our study. In general, compared to younger adults, we expected older adults to show lower performance in the learning of a complex visuospatial sequence, as well as lower scores in measures of cognitive functioning, related to working memory, attention and reasoning. Based on that, two hypotheses were postulated. First, we hypothesized that performance in the complex sequence learning task would be significantly related to a set of interrelated cognitive functions. Thus, by use of factor analysis, we aimed to identify independent subsets of cognitive functions that correlate with performance in the sequence learning task. Second, we predicted that age-related performance differences in perceptual sequence learning would be explained by age-related decrements in these task-relevant subsets of cognitive functions.

## Materials and Methods

### Participants

Eighteen younger (mean age ± SD: 23.61 ± 4.41 years, range: 18–32 years, 11 female) and 18 older (66.78 ± 6.09 years, 60–83 years, 10 female) adults participated in the current study. Younger participants were recruited from a pool of psychology students at the University of Tasmania and received either course credit or financial reimbursement for their participation. Older participants were recruited through an online advertisement and participated in a draw for gift vouchers as reimbursement. All participants gave written informed consent prior to participation, were right-hand dominant as assessed via the Edinburgh Handedness Inventory (Oldfield, 1971), had normal or corrected-to-normal vision, and no known neurological, cognitive or motor impairment that could impact their performance in the experiment. The experimental procedure was approved by the Human Research Ethics Committee (Tasmania) Network of the University of Tasmania and was in accordance with the principles stated in the Declaration of Helsinki.

### Procedure

#### Cognitive Assessment

All participants performed a series of cognitive tests to assess task-relevant dimensions of cognition, i.e., speed of processing, attention, visuospatial learning and memory, as well as spatial reasoning (see **Table 1**). The Matrix Reasoning Test (*MR*), a subtest of the Perceptual Reasoning Index of the Wechsler’s Adult Intelligence Scale IV (Wechsler et al., 2008) was used as a test of fluid intelligence with regard to spatial reasoning and abstract problem solving ability. Higher scores indicate a better ability to identify patterns and structures. Participants also completed the COGSTATE computerized cognitive test battery (COGSTATE Ltd^1^), which included the following subtests: Groton Maze Chase Test (*Chase*), Groton Maze Learning Test (*Maze*), Detection Task (*DET*), Identification Task (*IDN*), One Card Learning Task (*OCL*), One Back Task (*WM_1B*), Two Back Task (*WM_2B*), Continuous Paired Associate Learning Task (*CPAL*), and the Groton Maze Learning Test – Delayed Recall (*Maze_DR*). See **Table 1** for a description of the performance measures and their interpretation.

**Table 1:** Description of the cognitive tests assessed.

Test	Initial	Tested cognitive dimension	Performance Score	Interpretation
**Basic cognitive functions**				
*Groton Maze Chase Test*	Chase	Speed of visual processing	*Accuracy of performance:* Total number of correct moves made per second	Higher score = better performance
*Detection Task*	DET	Psychomotor function	*Speed of performance:* Mean of the log_10_ transformed reaction times for correct responses	Lower score = better performance
*Identification Task*	IDN	Attention	*Speed of performance:* Mean of the log_10_ transformed reaction times for correct responses	Lower score = better performance
**Visuospatial learning**				
*Continuous Paired Associate Learning Task*	CPAL	Paired-associate learning	*Errors:* Total number of errors	Lower score = better performance
*One Card Learning Task*	OCL	Visual learning	*Accuracy of performance:* Arcsine transformation of the proportion of correct responses	Higher score = better performance
**Executive function**				
*Groton Maze Learning Test*	Maze	Executive function	*Errors:* Total number of errors	Lower score = better performance
**Memory**				
*Groton Maze Learning Test – Delayed Recall*	Maze_DR	Memory	*Errors:* Total number of errors	Lower score = better performance
*One Back Task*	WM_1B	Working Memory	*Accuracy of performance:* Arcsine transformation of the square root of the proportion of correct responses	Higher score = better performance
*Two Back Task*	WM_2B	Working Memory	*Accuracy of performance:* Arcsine transformation of the square root of the proportion of correct responses	Higher score = better performance
**Spatial Reasoning**				
*Matrix Reasoning*	MR	Spatial reasoning and abstract problem solving	*Score:* Total number of correct answers	Higher score = better performance

#### Sequence Learning Task

**FIGURE 1:**
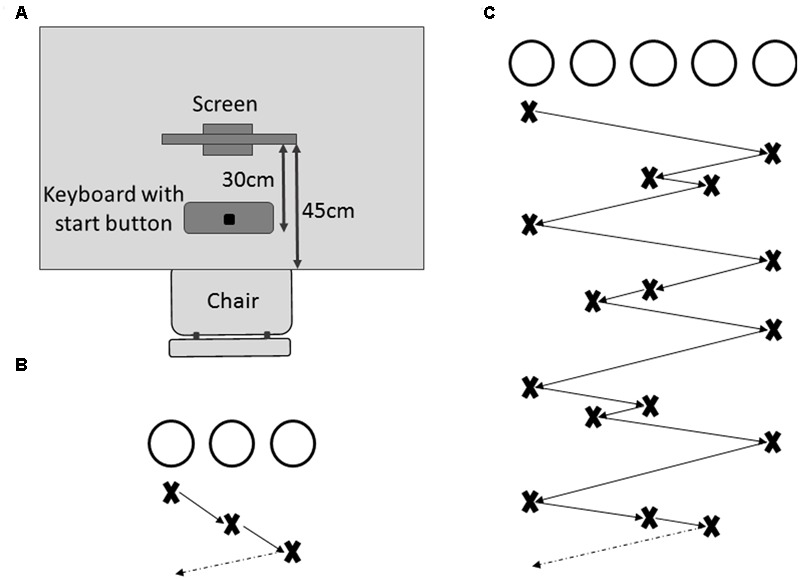
Experimental set-up. **(A)** Participants were seated on a chair in front of a table on which a touch screen was placed within reaching distance. In between the participant and the touch screen was a keyboard with the start button clearly marked. **(B)** Display of the practice sequence composed of three black, unfilled circles that illuminated in the order described by the crosses below the circles. **(C)** Display of the complex sequence to be learned in the complex sequence learning task (CSLT) with crosses and arrows describing the sequence order.

Following the cognitive assessment, all participants were familiarized with the experimental task, in which they had to learn a visuospatial multi-element sequence across repeated trials through observation. Participants were comfortably seated in front of a 23″ touch screen (Dell P2314Tt), placed on a table 45 cm from the edge, with a keyboard positioned 30 cm from the screen (see **Figure 1A**). All participants received written task instructions on the computer screen throughout the experiment, which was controlled through MATLAB R2011a (Mathworks, Natick, MA, United States), using the Psychophysics Toolbox extensions (Brainard, 1997; Kleiner et al., 2007). A perceptual visuospatial sequence learning task was chosen to minimize the impact of age-related declines in motor function on sequence learning performance (Ghilardi et al., 2003; Moisello et al., 2009).

##### Practice phase

All participants were familiarized with the task before the learning phase. Each practice trial was initiated by the participant pressing and holding down a designated start button (see **Figure 1A**). Immediately following the button press, three circles (2 cm diameter, 9 cm center-to-center distance) were presented on a white background (see **Figure 1B**). A simple 3-element sequence was then visually presented to the participants through sequential black filling of the circles (sequence order: left – middle – right, see **Figure 1B**). The first element in the sequence was color-coded through red filling of the circle. Each element was illuminated for 1 s with no delay between subsequent illuminations. After two complete runs of the sequence, the presentation stopped at a randomly chosen position during the third run, with the three unfilled circles remaining visible on the screen. After a random delay period of 1–2 s an acoustic signal (450 Hz, 0.2 s duration) informed participants to release the start button and point (i.e., reach and touch) to the circle on the touch screen that they predicted would be illuminated next in the sequence. Participants were required to release the start button within 750 ms following the acoustic signal. If the button release occurred too early (<100 ms after the acoustic signal) or too late (>750 ms), the trial was immediately aborted and an error message was presented (“You reacted too early. Please wait for the start tone next time.” or “You reacted too late. Please try to be faster next time.”), which reminded the participants of the time constraint related to their response. After completion of each pointing movement, participants were asked to indicate their confidence of having pointed to the correct circle on a 7-level Likert scale. Following the confidence rating, the next trial commenced. Participants were allowed to practice until they felt familiar with the procedure and initiated pointing movements consistently within the time constraints. None of the participants required more than 20 trials for familiarization.

##### Complex sequence learning task (CSLT)

In this task, participants performed five blocks of 15 trials each in which a complex 16-element sequence had to be learned, whereby five circles (2 cm diameter, 9 cm center-to-center distance) were presented on the touch screen. The sequence was developed such that no individual circle illuminated twice in succession and no more than two adjacent circles illuminated consecutively (see **Figure 1C**). The general procedure was identical to that described for the practice phase with sequence presentation being randomly stopped during the third run and participants indicating the predicted subsequent element in the sequence through a pointing movement. Between blocks, participants were given the opportunity to rest for a maximum of 5 min.

### Analysis

Data analysis was performed using customized MATLAB scripts. Only the first four experimental blocks were analyzed to mitigate possible fatigue effects that may have influenced performance in the fifth (final) block. First, for each block, the percentage of correct responses, i.e., the number of pointing movements to the correct element, was calculated. This percentage was used as a measure of *performance* in each block with changes in this measure across blocks being indicative of sequence learning. Participants whose performance was above or below 2.5 × SD from the mean were defined as outliers and excluded from further analyses. A mixed-factor repeated measures ANOVA was calculated on performance with age group as a between-subject factor, and block as within-subject factor. Paired-sample *t*-tests were conducted for *post hoc* analysis of the significant main effect of block. In addition, group differences in the measures of cognitive functioning were statistically analyzed using *t*-tests for independent samples.

Pearson’s correlations were calculated across all participants to identify relationships between performance in the CSLT and the measures of cognitive functioning. For the correlation analyses, performance in the first and fourth block was utilized. For cognitive tests that revealed significant correlations with performance in the CSLT, interrelations between these cognitive tests were analyzed by calculating Pearson’s correlations. To account for significant interrelations, an exploratory factor analysis was calculated. Following the recommendations of Osborne and Costello (2009), maximum likelihood method was utilized to extract independent factors (i.e., independent subsets of cognitive functions). Varimax rotation was applied to simplify interpretation of factor loadings. Subsequently, factor values were estimated using Bartlett’s maximum likelihood method (DiStefano et al., 2009) and Pearson’s correlations were calculated to identify relationships between the extracted factors and performance in the CSLT.

The critical level of statistical significance was set to α ≤ 0.05. The false discovery rate-approach was used to account for multiple testing in the correlation analyses (Benjamini and Hochberg, 1995). Greenhouse-Geisser corrections of the degrees of freedom were applied if the assumption of sphericity for the ANOVA was violated. Effect sizes (Cohen’s *d* and $\eta_{p}^{2}$) were calculated to aid in the interpretation of the magnitude of observed effects. In accordance with the recommendation of Sink and Stroh (2006), Cohen’s *d* ≥ 0.5 were considered as moderate effects and *d* ≥ 0.8 as large effects. Further, $\eta_{p}^{2}$ ≥ 0.06 were considered as medium effects and $\eta_{p}^{2}$ ≥ 0.14 as large effects. Similarly, correlations with *r* ≥ 0.5 were considered as moderate and *r* ≥ 0.8 as strong correlations. All statistical analyses were performed using SPSS Statistics 22 (IBM Corp., Armonk, NY, United States).

## Results

The results of group performances in the CSLT and in the cognitive tests will first be reported, after which the relationship between participants’ performance in the CSLT and the measures of cognitive functioning are described. Finally, the outcomes of the exploratory factor analysis and its effects on the age group analysis of CSLT performance will be reported. Two young participants had to be removed from the analysis because of the aforementioned outlier criterion. Results are based on the 87.5% of the trials in the CSLT in which the participants’ responses were initiated within the time constraints.

### Performance in the CSLT

**FIGURE 2:**
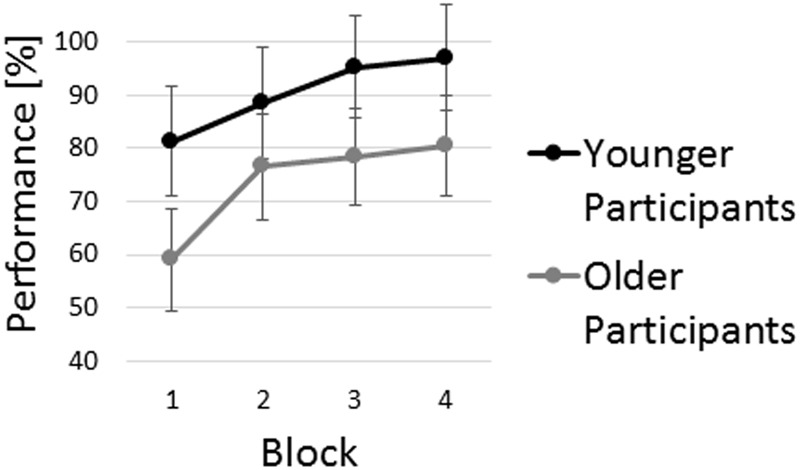
Age group analysis of sequence learning performance in the visuospatial sequence learning task. Average performance of both age groups is displayed. Error bars indicate 95% confidence intervals.

Older participants’ average performance was significantly lower than average performance of the younger participants (**Figure 2**), as indicated by a statistically significant main effect of group [*F*(1,32) = 7.70, *p* = 0.01, $\eta_{p}^{2}$ = 0.19]. Both age groups exhibited similar performance improvements, as indicated by the main effect of block [*F*(2.23,71.25) = 20.94, *p* < 0.001, $\eta_{p}^{2}$ = 0.40] and the non-significant interaction of block × group [*F*(2.23,71.25) = 1.32, *p* = 0.28, $\eta_{p}^{2}$ = 0.04]. *Post hoc* pairwise comparisons of single block performance revealed that first block performance was significantly worse than performance in the following three blocks (all *p* < 0.001).

### Cognitive Assessment

**FIGURE 3:**
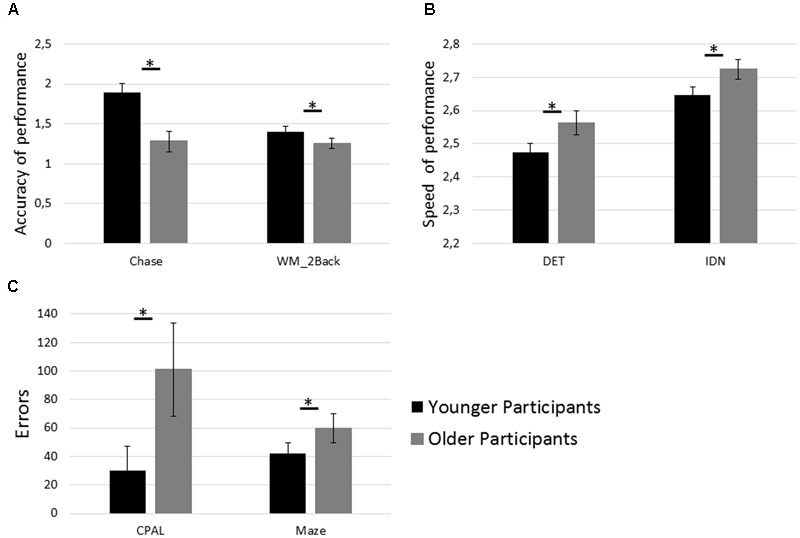
Age group differences in cognitive test performance. **(A)** Performance in the tests assessing speed of visual processing (Chase) and working memory capacity (WM_2Back). **(B)** Performance in the tests assessing psychomotor function (DET) and attention (IDN). **(C)** Performance in the tests assessing paired associate learning (CPAL) and executive function (Maze). Asterisks (^∗^) indicate *p* < 0.05 and error bars indicate 95% confidence intervals. Interpretation and calculation of performance measures are described in **Table 1**.

As expected, older participants exhibited lower performance in the subset of cognitive tests (see **Figure 3**) assessing basic cognitive functions: Chase [*t*(32) = 6.97, *p* < 0.001, *d* = 2.47], DET [*t*(32) = -4.10, *p* < 0.001, *d* = -1.45], IDN [*t*(32) = -4.21, *p* < 0.001, *d* = -1.49]; as well as visuospatial learning and memory: CPAL [*t*(32) = -3.96, *p* < 0.001, *d* = -1.39], WM_2Back [*t*(32) = 3.42, *p* = 0.002, *d* = 1.21] and executive function: Maze [*t*(32) = -2.91, *p* = 0.007, *d* = -1.03]. Three measures of cognitive functioning, namely OCL, WM_1B, and MR, did not show statistically significant differences between younger and older participants (*p* = 0.09, *p* = 0.38, and *p* = 0.38, respectively).

### Relation between CSLT Performance and Measures of Cognitive Functioning

Participants’ performance (i.e., percentage of correct responses) in the CSLT exhibited significant correlations with multiple cognitive tests, such that better scores in these tests were related to better performance in the CSLT (see **Table 2** for statistical values and **Table 1** for interpretation of scores that determine directionality of the correlation). Participants’ first and fourth block performance in the CSLT significantly correlated with their scores in five of the cognitive tests, namely Chase (*r* = 0.41, *p* = 0.02, and *r* = 0.46, *p* = 0.006, respectively), DET (*r* = -0.44, *p* = 0.009, and *r* = -0.46, *p* = 0.007), WM_2B (*r* = 0.42, *p* = 0.01, and *r* = 0.39, *p* = 0.02), Maze (*r* = -0.48, *p* = 0.004, and *r* = -0.40, *p* = 0.02), as well as CPAL (*r* = -0.43, *p* = 0.01, and *r* = -0.47, *p* = 0.005). Further, participants’ performance in the first block was significantly correlated with their score in the test of spatial reasoning ability (MR, *r* = 0.44, *p* = 0.009).

**Table 2:** Statistical values for the correlation analyses between cognitive test performance and learning performance at different stages of the CSLT, as well as for the correlation analysis between those cognitive functions of significant relevance for sequence learning performance.

	Sequence learning performance		Interrelations between cognitive functions				
	*Block 1*	*Block 4*	*DET*	*CPAL*	*Maze*	*WM_2Back*	*MR*
**Basic cognitive functions**							
*Chase*	*r* = 0.41, *p* = 0.02^∗^	*r* = 0.46, *p* = 0.006^∗^	*r* = -0.68, *p* < 0.001^∗^	*r* = -0.64, *p* < 0.001^∗^	*r* = -0.45, *p* = 0.008^∗^	*r* = 0.48, *p* = 0.004^∗^	*r* = 0.30, *p* = 0.08
*DET*	*r* = -0.44, *p* = 0.009^∗^	*r* = -0.46, *p* = 0.007^∗^		*r* = 0.53, *p =* 0.001^∗^	*r* = 0.36, *p =* 0.04	*r* = -0.27, *p* = 0.13	*r* = -0.38, *p* = 0.03^∗^
*IDN*	*r* = -0.34, *p* = 0.05	*r* = -0.37, *p* = 0.03					
**Visual-spatial learning**							
*CPAL*	*r* = -0.43, *p* = 0.01^∗^	*r* = -0.47, *p* = 0.005^∗^			*r* = 0.51, *p* = 0.002^∗^	*r* = -0.43, *p* = 0.01^∗^	*r* = -0.44, *p* = 0.009^∗^
*OCL*	*r* = 0.36, *p* = 0.04	*r* = 0.25, *p* = 0.16					
**Executive function**							
*Maze*	*r* = -0.48, *p* = 0.004^∗^	*r* = -0.40, *p* = 0.02^∗^				*r* = -0.38, *p* = 0.03^∗^	*r* = -0.63, *p* < 0.001^∗^
**Memory**							
*Maze_DR*	*r* = -0.34, *p* = 0.05	*r* = -0.26, *p* = 0.14					
*WM_1Back*	*r* = 0.09, *p* = 0.60	*r* = 0.22, *p* = 0.21					
*WM_2Back*	*r* = 0.42, *p* = 0.01^∗^	*r* = 0.39, *p* = 0.02^∗^					*r* = 0.13, *p* = 0.46
**Spatial Reasoning**							
*MR*	*r* = 0.44, *p* = 0.009^∗^	*r* = 0.27, *p* = 0.12					

*Asterisks indicate FDR-corrected statistically significant correlations.*

**FIGURE 4:**
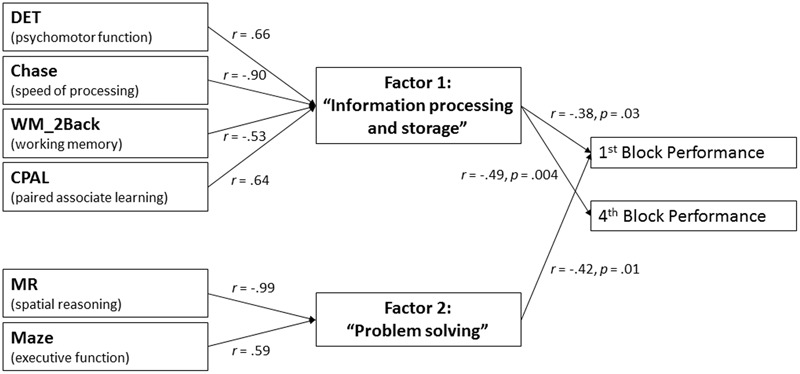
Factor analysis. Two independent subsets of cognitive functions related to either basic processing and storage of sequence information (Factor 1) or complex problem solving (Factor 2) were extracted. Note that while the six cognitive functions on the left hand side of the graph are interrelated, the two factors are mathematically independent of each other. Factor loadings of cognitive functions on the factors are displayed on the left set of arrows. For interpretation of directionality, please refer to **Table 1**. Significant correlations between factor scores and performance in the CSLT are displayed on the right set of arrows.

Each of the six cognitive functions significantly correlated with at least three other of these measures (see **Table 2**). Strongest correlations were found between Maze and MR (*r* = -0.63, *p* < 0.001), Chase and DET (*r* = -0.68, *p* < 0.001), as well as between Chase and CPAL (*r* = -0.64, *p* < 0.001). Thus, an exploratory factor analysis was calculated on these six measures of cognitive functioning after verifying its applicability by means of the Kaiser–Meyer–Olkin measure (equal to 0.76) and Bartlett test (*p* < 0.001). Adhering to the Kaiser-criteria, two factors were extracted (i.e., independent subsets of cognitive functions) that explained 71% of the total variance, i.e., giving a very good presentation of the interrelations between the measures of cognitive functioning. Further, the varimax rotation allowed the detection of four measures of cognitive functioning with high loadings on the first factor, namely DET, Chase, WM_2Back and CPAL (for all |r|≥ 0.53), with the remaining two measures, Maze and MR, loading highly on the second factor (for both |r|≥ 0.59, see **Figure 4**). Subsequently calculated Pearson’s correlations revealed that the participants’ placement on the first factor was significantly correlated with performance in the sequence learning task in Blocks 1 and 4 (*r* = -0.38, *p* = 0.03, and *r* = -0.49, *p* = 0.004, respectively), while participants’ placement on the second factor was significantly correlated only with Block 1 performance (*r* = -0.42, *p* = 0.01, see **Figure 4**). For both factors, higher factor scores correlated with lower performance in the CSLT.

### Relation of Age-Related Declines in CSLT Performance and Cognitive Functioning

Factor scores of the first factor were significantly higher in older than younger participants (mean ± SD: 0.80 ± 0.74 vs. -0.90 ± 0.48, respectively), as indicated by a statistically significant main effect of group [*t*(32) = -7.88, *p* < 0.001, *d* = -2.77], while factor scores of the second factor were not significantly different between younger and older adults [mean ± SD: -0.06 ± 0.91 vs. 0.05 ± 1.10, respectively, *t*(32) = -0.30, *p* = 0.76, *d* = -0.11]. Thus, in a final step the first factor was included as a covariate in the statistical analysis of performance differences in the CSLT between age groups. After inclusion of this factor in the analysis, the previously highly significant main effect of group turned out to be non-significant [*F*(1,31) = 0.26, *p* = 0.62, $\eta_{p}^{2}$ = 0.008], indicating no age-group differences in CSLT performance when controlling for inter-individual differences in the factor scores of factor 1. However, statistically significant differences were found for the main effect of block as well as the interaction of block × group [*F*(2.29,71.04) = 20.95, *p* < 0.001, $\eta_{p}^{2}$ = 0.40, and *F*(2.29,71.04) = 4.23, *p* = 0.02, $\eta_{p}^{2}$ = 0.12, respectively]. *Post hoc* analysis of the significant interaction effect revealed a trend towards significantly lower performance of older than younger participants in the first block [*F*(1,31) = 3.89, *p* = 0.06, $\eta_{p}^{2}$ = 0.11], and no performance differences thereafter (all *p* ≥ 0.82).

## Discussion

The aim of this study was to investigate the association between age-related differences in perceptual learning of a complex visuospatial sequence and age-related declines in cognitive functioning. Younger and older participants were tested on various cognitive functions using a standardized test battery. Participants then performed a perceptual visuospatial sequence learning task. Performance in the complex sequence learning task (CSLT) was analyzed and related to participants’ cognitive functioning.

### Age-Related Performance Differences in Sequence Learning and Cognitive Functioning

In line with previous research, significant differences in sequence learning performance were observed between age groups (see **Figure 3**). While both age groups improved performance across blocks, the between-group performance difference remained significant even within the fourth block. This finding replicates previous studies on age-related performance differences in perceptual and motor learning tasks (Ghilardi et al., 2003; Bo et al., 2009; Ghisletta et al., 2010; Panzer et al., 2011). Further, in line with previous research on cognitive aging (Rabbitt and Lowe, 2000) various cognitive functions were shown to be negatively affected by aging. These included basic cognitive functions, i.e., speed of processing, psychomotor function, and attention; cognitive functions related to memory and learning, i.e., working memory, and paired-associate learning; as well as executive function. The strong accordance of our data with previous studies on age-related performance differences in sequence learning and cognitive functioning provided the basis for the further analyses examining the contribution of independent cognitive factors to sequence learning in the two age groups.

### Relation between Sequence Learning Performance and Cognitive Functioning

Performance in the CSLT significantly correlated with cognitive functions that have previously been shown to relate to perceptual and motor sequence learning, particularly working memory and spatial reasoning (Unsworth and Engle, 2005; Bo and Seidler, 2009; Bo et al., 2009). In addition, other cognitive functions, namely executive function, paired-associate learning, speed of processing and psychomotor function, were also found to significantly correlate with sequence learning performance, lending empirical support to our first hypothesis that multiple cognitive functions significantly influence performance in a complex sequence learning task.

Importantly, all of these cognitive functions were also interrelated with each other. By use of exploratory factor analysis, we were able to unravel these interrelations and extract two independent subsets of cognitive functions (i.e., factors, see **Figure 4**), both of which correlated with specific aspects of CSLT performance. The first factor was primarily composed of cognitive functions related to basic processing and storage of sequence information, i.e., speed of processing, psychomotor function, working memory capacity and paired associate learning. This factor significantly correlated with performance in both the first and last block of the CSLT, suggesting that the ability to effectively process and store sequence information is of relevance for performance in the CSLT at both an early and later stages of learning. In contrast, the second factor, which was composed of executive function and spatial reasoning ability, i.e., cognitive abilities relevant for complex problem solving, only correlated with *first block* performance in the CSLT, suggesting that the ability to find a solution for abstract problems, i.e., deciding on what is the next in a sequence of elements, determines performance only during *early exposure* with the complex sequence. We suggest, therefore, that once the sequence became more familiar, deciding on the next element in the sequence no longer required complex problem solving functions. Consequently, problem solving abilities are of lesser importance for task performance at this later stage of learning. Last, inter-individual differences in spatial reasoning ability in younger adults have been previously shown to correlate with inter-individual differences in the degree of learning in an explicit motor sequence learning task (Unsworth and Engle, 2005). The current results suggest that this finding can be extended to an early stage of *perceptual* sequence learning.

### Influence of Age-Related Declines in Cognitive Functioning on Sequence Learning

The two independent subsets of cognitive functions that distinctively related to performance in the CSLT also showed different sensitivity to aging. Significant age group differences were observed for the first factor, related to the processing and storage of sequence information, whereas no age-related differences were evident for the second factor, related to complex problem solving. Similar to our findings, Charness (1981), in a study on age-related performance differences in chess players, found differences in the ability to encode and retrieve information but not problem solving between younger and older chess players. While age-related decrements in information processing and storage are well-documented (Nettelbeck and Rabbitt, 1992; Rabbitt and Lowe, 2000; Salthouse, 2000), empirical evidence on age-differences in problem solving abilities is more inconclusive. Thornton and Dumke (2005), for example, in their meta-analysis of 28 studies on everyday problem solving and decision making, did not find empirical support for preserved problem solving abilities in older adults. In contrast, Blanchard-Fields et al. (2007) found empirical evidence for preserved abilities in older adults in the context of interpersonal problem solving. Preservation in problem solving abilities is often attributed to a shift to simpler or more efficient problem solving strategies in older adults (Geary and Wiley, 1991; Blanchard-Fields et al., 2007; Mata et al., 2007; Lemaire, 2010). Ambiguity about age-sensitivity exists also with regard to the two cognitive functions underlying problem solving: executive function and spatial reasoning ability. While we did not find a significant age group difference in spatial reasoning ability, age-related decrements were evident for executive function. In contrast, Rabbitt and Lowe (2000) found spatial reasoning ability but not executive function to be age-sensitive. Overall, our results lend support to the existing empirical evidence that the set of cognitive functions related to problem solving mediates performance differences in a range of cognitive and everyday tasks *independent* of age.

Importantly, when including the first factor as a covariate in the statistical analysis of performance differences in the CSLT to account for age-related differences in the ability to process and store sequence information, the significant age group effect was abolished. In addition, the interaction of block × age group was now observed to be statistically significant with *post hoc* analysis indicating a lower performance of older than younger adults in the first block, but not thereafter. This suggests that a substantial component of the observed age-related performance differences in the CSLT can be attributed to performance differences in the subset of cognitive abilities related to the basic processing and storage of sequence information.

### Future Research Directions

Future research should aim to investigate whether behavioral differences in perceptual sequence learning are also apparent at the neurophysiological level. This would increase our understanding of age-dependent changes in cortical activity mechanisms that correlate with the exhibited behavioral declines. A study by Moisello et al. (2013), utilizing an explicit visual sequence learning task, provides interesting first insights into performance related differences in resting state oscillatory brain activity. Further, the application of an “individual difference approach” (Charness, 1981; Salthouse, 2016) might represent a valuable approach to further our understanding of within-age group differences in sequence learning performance and their relation to cognitive functioning.

## Conclusion

The current findings indicate that age-related differences in the performance of a perceptual visuospatial sequence learning task are determined by age-related declines in a set of cognitive abilities, which correlated with task performance at both early and late stages of learning. This set of cognitive functions relates to the basic processing and storage of sequence information, and includes speed of visual processing and psychomotor function, as well as working memory and paired-associate learning. A second, independent set of cognitive functions, related to complex problem solving and including executive function and spatial reasoning ability, was found to correlate with performance in the sequence learning task only at an early stage of learning, independent of age. In sum, the results suggest that age-related declines in perceptual visuospatial sequence learning are mediated by age-related declines in information processing and storage, while problem solving abilities mediate performance differences independent of age.

## Author Contributions

All authors of this study contributed substantially to its conception, the interpretation of data, as well as drafting and revising earlier versions of the manuscript. In addition, MK was responsible for data acquisition and analysis. MK, MH, RP, and JS all approved the version to be published and agreed to be accountable for all aspects of the work.

## Conflict of Interest Statement

The authors declare that the research was conducted in the absence of any commercial or financial relationships that could be construed as a potential conflict of interest.

## Footnotes

1 www.cogstate.com
